# From the President’s Desk – Part 1, 2022

**DOI:** 10.4102/safp.v64i1.5465

**Published:** 2022-01-31

**Authors:** Robert Mash

**Affiliations:** 1 Division of Family Medicine and Primary Care, Faculty of Medicine and Health Sciences, Stellenbosch University, Cape Town, South Africa; 2 South African Academy of Family Physicians, Durbanville, South Africa

This has been a busy year for the Academy and I am excited about a number of our initiatives that will produce successful results in 2022. Over the last few weeks, the Wonca World Conference was held virtually in Abu Dhabi and although our bid to host the conference in 2025 was not successful, a number of South African family physicians were honoured. Prof. Gboyega Ogunbanjo and Prof. Sam Fehrsen received the Fellowship of Wonca posthumously. Prof. Pierre de Villiers and Prof. Khaya Mfenyana were awarded a Direct Lifetime Honorary Membership of Wonca for their contribution to the discipline. Prof. Shabir Moosa was voted in as a Member at large and part of the global executive.

Plans are already underway to have our National Family Practitioners Conference in Cape Town on 19–20 August 2022. Please diarise these dates! We plan a hybrid conference with most people attending in person, but with opportunities also for virtual attendance. We really hope that we can see each other face-to-face in 2022. The proposed theme of the conference is ‘Bouncing back – resilience in the face of change’. The theme speaks about learning to live with the coronavirus as we bounce back from the disruption of the coronavirus disease 2019 (COVID-19) pandemic in 2020 and 2021. The pandemic has required great resilience from family doctors and all healthcare workers in the face of continual change and adaptation. Resilience has been needed at personal, professional and organisational levels. We have lost ground against some of the other health priorities such as human immunodeficiency virus (HIV), tuberculosis (TB), diabetes and hypertension. In addition we learnt valuable lessons on how to harness technology to improve patient care. The conference will also focus on meeting your learning needs that were identified in the survey we conducted with our members during 2021.

The Academy has just launched a survey of our members in private practice and from the responses we will establish a private sector forum under the leadership of Dr Sheena Mathew. The forum will guide us in our advocacy for the needs of the private sector family physicians. The private sector has discriminated against specialists in family medicine for too long in terms of both remuneration and scope of practice. We hope that 2022 will be a year when we start to advocate more effectively for our members in the private sector. I encourage you to join the private sector forum.

Likewise, I am delighted about the emergence of the Next-5 to look after the needs of newly qualified family physicians as they transition from registrar to family physician. We hope that this special interest group will grow and develop in 2022 under the leadership of Drs Chantelle van der Bijl and Arun Nair.

As I look towards 2022, I am excited about the Academy providing an e-portfolio for the training of registrars. The SCORION e-portfolio was developed by Parantion, a company in the Netherlands, and the development costs covered by the Dutch government. The e-portfolio was piloted with registrars at Stellenbosch University during 2021 under the leadership of Prof. Louis Jenkins and Dr Ts’epo Motsohi. In 2022, the Academy will act as an umbrella body to make the e-portfolio available to all training programmes in South Africa. We will be the first discipline in South Africa to share one national electronic portfolio that supports workplace-based learning and assessment.

Finally, the Academy is preparing a new position paper on family physicians in the South African health system and we hope not only to publish this, but also to engage actively with policymakers in 2022. It is time for human resources for health policy nationally and in all provinces to recognise the important contribution of family physicians to strengthening primary health care and district hospitals.

